# Did the COVID-19 Pandemic Influence the Awareness About Vitamin D Among the General Population in Jeddah, Saudi Arabia?

**DOI:** 10.7759/cureus.50117

**Published:** 2023-12-07

**Authors:** Sahar S Othman, Majed S Almalki, Ali Suliman Alblwi, Abdullah M Alqarni, Hassan Hussein Alharbi, Abdualaziz Adel Gizani, Abdualaziz Matar Alharthi, Badr Nasraldeen Alareeshe, Salem Faisal Alsaedi

**Affiliations:** 1 Department of Family Medicine, Faculty of Medicine, King Abdulaziz University, Jeddah, SAU; 2 Medical School, Faculty of Medicine, King Abdulaziz University, Jeddah, SAU

**Keywords:** awareness, pandemic, jeddah, covid-19, vitamin d

## Abstract

Background

Vitamin D deficiency has been a major health concern over the last decade. With the coronavirus disease 2019 (COVID-19) pandemic, health officials and social media have stressed the importance of vitamin D and its role in immune systems. This research focused on the level of vitamin D awareness in Jeddah after the pandemic in 2022.

Methodology

A cross-sectional study was conducted among the adult general population in Jeddah, Saudi Arabia. Data were obtained through an electronically distributed questionnaire designed to collect information on self-reported knowledge questions about vitamin D, which was previously validated and used in a similar study on the same population before the pandemic. The validated questionnaire included sociodemographic characteristics and questions assessing the knowledge about vitamin D. Ethical approvals were obtained from the Biomedical Research Ethics Committee, Faculty of Medicine, King Abdulaziz University, Jeddah, Saudi Arabia.

Results

Of the 385 total participants, 198 (51.4%) were aged 18-28 years, 331 (86%) were of Arab ethnicity, 289 (75.1%) had a university degree, 86 (22.3%) had completed high school, 186 (48.3%) were married, and 197 (51.2%) had no children. The overall mean knowledge score was 67.12%, and the mean knowledge score for vitamin D benefits was 73.51%. The mean knowledge score for vitamin D sources was 51.53%, and the mean knowledge score for toxicity was 86.49%. When comparing knowledge scores based on demographic variables, only a few variables were significant. Regarding vitamin D awareness following the COVID-19 pandemic, most participants (54.55%) believed the pandemic had affected or increased their vitamin D awareness. Approximately 52.85% had used vitamin D supplements before the pandemic, and 53.25% were currently using vitamin D supplements.

Conclusions

In general, compared to pre-COVID-19 studies conducted in Saudi Arabia, this study revealed a greater understanding of vitamin D. Participants who had completed high school or more displayed a higher level of knowledge than other groups. It is still recommended that primary care physicians educate their patients and families about the benefits of vitamin D, the effects of its deficiency, and its toxicity.

## Introduction

Today’s world is impacted by unhealthy lifestyles and behaviors that can lead to a variety of conditions, including vitamin D deficiency and insufficiency [1]. Vitamin D deficiency is considered one of the most common undetected medical conditions worldwide [2]. Globally, studies have shown a prevalence rate of vitamin D deficiency ranging from 30% to 80% [3,4]. In Saudi Arabia, the prevalence rate of vitamin D deficiency was found to be as high as 81% in a study published in 2017 [5], which reviewed the literature, epidemiological studies, and clinical trials related to this condition conducted in Saudi Arabia between the years 2011 and 2016 [5,6].

Despite the vitamin D deficiency epidemic, support for early screening is absent in addition to the high cost of 25(OH)D blood level measurement [7]. However, testing for vitamin D may be of benefit for those at higher risk such as malnutrition and bowel conditions that affect absorption, as well as those with vitamin D deficiency-related findings on imaging or laboratory testing (e.g., fragility and low bone density, or high parathyroid hormone level) [8]. There is currently no universal agreement on the recommended vitamin D dosage [9]. In most healthy individuals, 800 IU of vitamin D daily appears to be adequate to attain a goal 25(OH)D level of at least 50 nmol/L (or 20 ng/mL), whereas 2,000 IU appears to be sufficient to achieve a level of at least 75 nmol/L (or 30 ng/mL) [10]. Factors such as an individual’s age, weight, health concerns, dietary habits, and cultural and socioeconomic status may have led to vitamin D recommendations being more clinically based rather than following a fixed national guideline [11]. To elaborate on why vitamin D deficiency is a major concern, there is a need to highlight the well-known physiologic effects of vitamin D on the innate and adaptive immune systems [12]. Vitamin D decreases the risk of viral infection, bone weakness, osteoporosis, and mortality in a variety of ways [13].

In the early months of the coronavirus disease 2019 (COVID-19) pandemic, vitamin D was favored as a preventive and promising supportive therapy [14]. Severe vitamin D deficiency was shown to be a predictor of mortality among COVID-19 patients in Saudi Arabia [15]. A study done in Riyadh, with a sample size of 439 confirmed COVID-19 cases, found that the most common comorbidity (74.7%) was vitamin D deficiency [16]. In another study among 222 hospitalized patients screened for COVID-19, vitamin D deficiency was present in 75% of all patients, and the levels were significantly lower among SARS-CoV-2 (+) than SARS-CoV-2 (-) patients [17]. Another multi-center study among Arab Gulf residents found that the vitamin D levels of confirmed COVID-19-positive patients were much lower than those who tested negative, concluding that large population-based studies should be conducted to assess the protective effects of vitamin D supplementation against COVID-19 [18].

In regards to assessing knowledge about vitamin D, international studies from Great Britain, Lybia, and Sudan have shown a decent increase in vitamin D knowledge and to some extent its benefits, but there is a lack of knowledge regarding vitamin D resources and outcomes of its deficiency, combined with a poor attitude toward sun exposure [19]. On a national level, a cross-sectional study was conducted in 2017 among the population of Jeddah, Saudi Arabia, to determine the awareness about vitamin D. It revealed insufficient recognition of vitamin D deficiency among participants and a significant association between the level of knowledge about vitamin D and the education level of participants. However, there was a high level of awareness regarding the benefits of vitamin D. The study also showed that participants who did not have children had the highest knowledge about benefits [20].

Another cross-sectional study among Saudi people in Riyadh demonstrated poor knowledge and practices regarding vitamin D deficiency. The findings highlighted the significance of implementing public ongoing education programs to assist in building greater awareness and information about the benefits of vitamin D [21].

It is unknown whether the COVID-19 pandemic and the associated health education has increased the public’s knowledge about vitamin D. Now after more than two years since the beginning of the COVID-19 pandemic, we aim to measure the awareness about vitamin D among the general population in Jeddah and compare it to the pre-pandemic findings.

## Materials and methods

Study design and setting

A non-interventional, cross-sectional study was conducted among the general population of Jeddah, Saudi Arabia after the COVID-19 pandemic in 2022.

Sample size and population

The sample size was calculated using the single proportion equation in the Raosoft software package. Based on the assumption that the rate of awareness was 50% and a margin of error of 5% at the 95% confidence level, the required sample size was 385.

The targeted individuals were residents of Jeddah city, of both genders, with the exclusion of those aged less than 18 years.

Data collection and definition of variables

Data were obtained through a self-administered questionnaire designed to collect information on self-reported knowledge about vitamin D. The snowball sampling technique was employed, and the survey was distributed online in August 2022.

A validated questionnaire from a similar previously published study [22] on the same population before the pandemic was used to collect demographic variables and data on vitamin D knowledge. The study was published in an open-access journal, which permits the reuse and distribution of any parts with a proper citation.

The validated questionnaire used in a previous study was divided into two parts, namely, sociodemographic (age, gender, nationality, highest educational qualification, and the number of children), and information regarding knowledge about vitamin D (benefits, deficiency, resources, and toxicity). In the knowledge section questions, the score was given as follows: for each correct answer, 1 point was given, and for an incorrect answer, 0 points were given, with the overall score being 14.

A third section about the impact of COVID-19 on vitamin D awareness was added by the researchers after reviewing relevant literature [13,23].

Data entry and data analysis

The 2016 version of Microsoft Excel (Microsoft Corp., Redmond, WA, USA) was used for data entry, and the SPSS version 21 (IBM Crop., Armonk, NY, USA) was used for data analysis. Qualitative data were described using frequencies and percentages. Quantitative data were described using means and standard deviation. Knowledge scores were compared across demographic groups using an independent t-test or analysis of variance for variables with more than two groups. A 95% confidence level and a 5% margin of error were guaranteed when describing quantitative variables. Statistical significance was determined at p-values <0.05.

Ethical considerations

Ethical approval for this study was obtained on August 1, 2022, from the Biomedical Research Ethics Committee, Faculty of Medicine, King Abdulaziz University, Jeddah, Saudi Arabia (reference number: 385-22). Written informed consent was obtained from each participant before filling out the questionnaire, and all data were kept anonymous and confidential.

## Results

Of 385 total participants, 198 (51.4%) were aged 18-28 years, and 331 (86%) were of Saudi nationality. Regarding education, 289 (75.1%) had a university degree, and 86 (22.3%) had completed high school. Overall, 186 (48.3%) participants were married, and 197 (51.2%) had no children (Table 1).

**Table 1:** Participant characteristics.

Variable	N	%
Age		
18–28	198	51.43
29–39	66	17.14
40–50	64	16.62
51–60	44	11.43
More than 60	13	3.38
Nationality		
Saudi	331	85.97
Non-Saudi (Egyptian, Iraqi, Sudanese, and Yemen)	54	14.03
Education		
University	289	75.06
High school	86	22.34
Intermediate	6	1.56
Elementary	4	1.04
Marital status		
Married	186	48.31
Single	180	46.75
Divorced	11	2.86
Widowed	8	2.08
Have children		
Yes	188	48.83
No	197	51.17

Awareness regarding the benefits of vitamin D is shown in Table 2. The majority of the participants agreed that vitamin D is important in the maintenance of calcium and phosphates, and thought that vitamin D is used to treat bone disease and rickets (95.58%). Overall, 83.90% of participants believed that vitamin D helps strengthen immunity, and 74.55% thought that it strengthens muscles (Table 2).

**Table 2:** Awareness regarding the benefits of vitamin D.

Do you think vitamin D is used to treat bone disease and rickets		
Variable	N	%
Yes	368	95.58
No	17	4.42
Do you think vitamin D is important in the maintenance of calcium and phosphates		
Yes	352	91.43
No	33	8.57
Do you think vitamin D is important in the maintenance of bone and teeth		
Yes	368	95.58
No	17	4.42
Do you think Vitamin D helps to strengthen immunity		
Yes	323	83.90
No	62	16.10
Do you think vitamin D helps to strengthen muscles		
Yes	287	74.55
No	98	25.45

Awareness of the sources of vitamin D is shown in Table 3. The majority of participants did not believe that vitamin D is exclusive to animal meat, fruit, and vegetables (69.61%). When they were asked if vegetarians are more likely to have vitamin D deficiency, 51.89% answered yes. On the other hand, only 43.38% thought that a free-fat diet can cause vitamin D deficiency. Overall, 53.51% also thought that frequent sun exposure can lead to vitamin D poisoning. Approximately 94.55% thought that sun exposure encourages vitamin D production in the skin, and 76.10% agreed that people residing in cloudy areas are more prone to vitamin D deficiency. Moreover, 56.62% disagreed that sunscreens are a cause of vitamin D deficiency. More than two-thirds of our participants (70.65%) answered no when they were asked if darker skin is more prone to vitamin D deficiency than fairer skin (Table 3).

**Table 3:** Awareness regarding sources of vitamin D.

Variable	N	%
Do you think Sun exposure encourages vitamin D production in the skin		
Yes	364	94.55
No	21	5.45
Do you think that vitamin D is found in animal meat but not in vegetables and fruits		
Yes	117	30.39
No	268	69.61
Do you think that people residing in cloudy areas are more prone to vitamin D deficiency		
Yes	293	76.10
No	92	23.90
Do you think frequent sun exposure does not lead to vitamin D poisoning		
Yes	179	46.49
No	206	53.51
Do you think the use of sunscreens may be a cause of vitamin D deficiency		
Yes	155	40.26
No	230	59.74
Do you think a fat-free diet may be a cause of vitamin D deficiency		
Yes	167	43.38
No	218	56.62
Do you think dark skin is more prone to vitamin D deficiency than fairer skin		
Yes	113	29.35
No	272	70.65
Do you think vegetarians are more likely to have vitamin D deficiency than non-vegetarians		
Yes	199	51.69
No	186	48.31

Awareness of vitamin D toxicity is shown in Table 4. Approximately 86.49% were aware that increased level of calcium in the blood exposes them to health problems (Table 4).

**Table 4:** Awareness regarding consequences of vitamin D toxicity.

Variable	N	%
Do you think the increased level of calcium in the blood exposes you to health problems?		
Yes	333	86.49
No	52	13.51

Table 5 shows the mean score of knowledge about vitamin D based on the first three variables (Tables, 2, 3, and 4). The overall mean knowledge score was 9.40 ± 2.262 (67.12%). The mean knowledge score for benefits was 4.1 ± 0.94 (73.51%), while the mean knowledge score for sources was 4.12 ± 1.78 (51.53%). The mean knowledge score for toxicity was 0.86 ± 0.34 (86.49%).

**Table 5:** Mean score of knowledge about vitamin D based on the first three variables.

Variables	Mean ± SD	Range (minimum-maximum)	Total	%
Benefits	4.1 ± 0.94	0–5	6	73.51
Sources	4.12 ± 1.78	0–5	8	51.53
Toxicity	0.86 ± 0.34	0–1	1	86.49
Overall	9.40 ± 2.26	0–11	5	67.12

When comparing knowledge scores based on demographic variables, only a few variables were significant. There was no significant difference among age groups (Table 6) or marital status (Table 7) for any of the knowledge domains (benefits, source, toxicity, and overall). In contrast, when comparing education levels, there was a significant difference in overall knowledge scores. High school participants had the highest score, followed by those with a university degree (p = 0.04). No significant difference was found among education levels for the other domains (benefits, sources, and toxicity) (Table 8). Lastly, when examining participants’ knowledge scores based on having children, the results revealed a significant difference in benefits, toxicity, and overall knowledge scores. Participants who did not have children had the highest score for benefits, toxicity, and overall (p = 0.009, 0.004, and 0.03, respectively). No significant difference was found regarding knowledge about vitamin D sources (Table 9).

**Table 6:** Knowledge score by age. P < 0.05 was considered significant.

Variable	Mean ± SD	P-value
Benefits		
18–28	4.30 ± 1.031	
29–39	4.56 ± 1.025	
40–50	4.58 ± 0.662	0.071
51–60	4.41 ± 1.187	
More than 60	4.54 ± 0.776	
Sources		
18–28	4.17 ± 1.854	
29–39	3.70 ± 1.945	
40–50	4.34 ± 1.556	0.74
51–60	4.23 ± 1.523	
More than 60	4.08 ± 1.656	
Toxicity		
18–28	0.85 ± 0.354	
29–39	0.92 ± 0.267	
40–50	0.84 ± 0.366	0.19
51–60	0.86 ± 0.347	
More than 60	0.85 ± 0.376	
Overall		
18–28	9.32 ± 2.349	
29–39	9.18 ± 2.372	
40–50	9.77 ± 1.771	0.56
51–60	9.50 ± 2.348	
More than 60	9.46 ± 2.222	

**Table 7:** Knowledge score by marital status. P < 0.05 was considered significant.

Variable	Mean ± SD	P-value
Benefits		
Married	4.51 ± 1.004	
Single	4.31 ± 0.952	
Divorced	4.73 ± 0.467	0.07
Widowed	4.00 ± 1.773	
Sources		
Married	4.13 ± 1.770	
Single	4.11 ± 1.824	
Divorced	4.45 ± 1.864	0.81
Widowed	3.63 ± 1.408	
Toxicity		
Married	0.87 ± 0.342	
Single	0.86 ± 0.347	0.39
Divorced	1.00 ± 0.000	
Widowed	0.75±0.463	
Overall		
Married	9.51 ± 2.221	
Single	9.28 ± 2.267	
Divorced	10.18 ± 1.834	0.48
Widowed	8.38 ± 3.335	

**Table 8:** Knowledge score by education. P < 0.05 was considered significant.

Variable	Mean ± SD	P-value
Benefits		
University	4.49 ± 0.847	
High school	4.55 ± 0.762	
Intermediate	4.67 ± 0.516	0.28
Elementary	4.25 ± 0.500	
Sources		
University	4.24 ± 1.789	
High school	3.60 ± 1.625	0.08
Intermediate	4.67 ± 1.211	
Elementary	4.75 ± 2.43	
Toxicity		
University	0.89 ± 0.309	
High school	0.86 ± 0.349	
Intermediate	0.83 ± 0.408	0.07
Elementary	0.75 ± 0.500	
Overall		
University	9.63 ± 2.233	
High school	9.01± 1.779	
Intermediate	10.17 ± 1.602	0.04
Elementary	9.75 ± 3.304	

**Table 9:** Knowledge score by parental status. P < 0.05 was considered significant.

Variable	Mean ± SD	P-value
Benefits		
Yes	4.54 ± 0.927	
No	4.28 ± 1.040	0.009
Not married	4.31 ± 0.952	
Sources		
Yes	4.11 ± 1.722	
No	4.13 ± 1.852	0.63
Not married	4.11 ± 1.824	
Toxicity		
Yes	0.86 ± 0.346	
No	0.87 ± 0.339	0.004
Not married	0.86 ± 0.347	
Overall		
Yes	9.52 ± 2.143	
No	9.28 ± 2.369	0.03
Not married	9.28 ± 2.267	

Regarding awareness of vitamin D after the COVID-19 pandemic, most participants thought that the pandemic had increased their awareness of vitamin D (54.55.%). Approximately 52.85% had used vitamin D supplements before the pandemic, and 53.25% stated that they currently used vitamin D supplements (Table 10).

**Table 10:** Awareness after the COVID-19 pandemic.

Variable	N	%
Are you using a vitamin D supplement?		
Yes	205	53.25
No	180	46.75
Do you think the pandemic has affected or increased your awareness of vitamin D?		
Yes	210	54.55
No	175	45.45
If you answered “Yes,” have you used the vitamin D supplement before the pandemic?		
Yes	111	52.85
No	99	47.41

## Discussion

The rationale for picking this period is the changes observed in people’s thinking and behavior regarding health after the COVID-19 pandemic, specifically on some vitamin supplements including vitamin D. In the early months of the pandemic, there was considerable focus on vitamin D from major public health figures and national health organizations to the extent that it was favored as a preventive and promising supportive therapy due to its well-established involvement in immunity and disease prevention. In addition, there was extensive media coverage in which many social media influencers stated that they have started taking vitamin D supplements since the start of the pandemic and encouraged their followers to do the same [23-25].

The results revealed that over two-thirds of the total participants had an appropriate level of knowledge regarding vitamin D benefits, contrary to global findings where there is a low level of awareness of vitamin D that would be considered a contributing factor to high levels of vitamin D deficiency worldwide. There was also a high level of vitamin deficiency according to a large adult cohort study in the United Kingdom, which revealed that up to 46.6% of white British adults had vitamin D insufficiency, with 15.5% having severe deficiency during winter or spring [26]. Similarly, a previous Chinese study conducted via telephone interviews showed a low level of awareness of the sources and role of vitamin D among Chinese women living in Hong Kong [27]. Considering that vitamin D is not typically a part of daily meals, the main source of vitamin D comes from exposure to sunlight, making it often necessary to prescribe vitamin D supplements to persons who have limited sun exposure or decreased cutaneous synthesis, such as in the elderly [28].

Moreover, on a national level, previous findings were similar to international studies. A Saudi Arabian study found that 40% of women who used primary healthcare facilities in Al-Ahsa were unaware of the advantages of vitamin D supplementation in their infants [29].

Locally, in the city of Jeddah, a study conducted before the pandemic in 2017 by Alamoudi et al. found that the overall mean knowledge score of participants was 39.3%, with mean knowledge scores for benefits, resources, and toxicity being 60%, 35%, and 30%, respectively. With these numbers in mind, our study showed a remarkable increase in mean knowledge scores in 2022 compared to the previous study. There was an increase of 27.82% (from 39.3% to 67.12%) in the overall knowledge score, an increase of 13.51% in the benefits knowledge score (from 60% to 73.51%), 16.53% in the resources knowledge score ( from 35% to 51.53%), and more than 50% increase in the toxicity knowledge score (from 30% to 86.49%).

From the previous comparison we can see how the awareness of the public population in Jeddah has improved since the pandemic, we believe this is due to the heavy media coverage of vitamins and nutrition in general during this period. The majority of our sample (53.25%) acknowledged using vitamin D supplements, and about 47.41% of the participants started using them after the pandemic. In addition, more than half of the participants (54.55%) believed that the pandemic had increased their awareness of vitamin D. Significant disparities in knowledge scores for education level and having children were found when sociodemographic groups were compared. High school students and people without children demonstrated a higher level of knowledge than other groups. However, no marked differences were noted regarding age, gender, and marital status.

Study limitations

This study has some limitations that need to be addressed. First, as in all observational studies, only association can be observed but no causal relationships can be established. In addition, sunlight avoidance was not investigated, but it can be speculated through the common indoor lifestyle in Saudi Arabia. Finally, as the survey was conducted in only one city (Jeddah), the results cannot be generalizable to the entire population of the Kingdom of Saudi Arabia.

## Conclusions

This study investigated how the COVID-19 pandemic influenced the awareness of vitamin D among the general population in Jeddah, Saudi Arabia. Generally, we found an improved awareness regarding vitamin D when compared with pre-COVID-19 studies from Saudi Arabia. Participants with high school education and higher showed a better level of knowledge than other groups. Therefore, we believe that educational efforts directed at certain demographics will raise awareness. Furthermore, primary healthcare doctors should emphasize and explain to the patients and their families the importance of vitamin D and the consequences of its deficiency as well as its toxicity whether by adding extra time in the clinic to educate them or by sharing simple medical information in the media.
